# Intersectional analysis of inequalities in self-reported breast cancer screening attendance using supervised machine learning and PROGRESS-Plus framework

**DOI:** 10.3389/fpubh.2023.1332277

**Published:** 2024-01-05

**Authors:** Núria Pedrós Barnils, Benjamin Schüz

**Affiliations:** Institute for Public Health and Nursing Research, University of Bremen, Bremen, Germany

**Keywords:** healthcare access, breast cancer screening, health inequalities, intersectionality, decision trees, supervised machine learning, PROGRESS-Plus framework, Spain

## Abstract

**Background:**

Breast cancer is a critical public health concern in Spain, and organized screening programs have been in place since the 1990s to reduce its incidence. However, despite the bi-annual invitation for breast cancer screening (BCS) for women aged 45–69, significant attendance inequalities persist among different population groups. This study employs a quantitative intersectional perspective to identify intersectional positions at risk of not undergoing breast cancer screening in Spain.

**Methods:**

Women were selected from the 2020 European Health Interview Survey in Spain, which surveyed the adult population (> 15 years old) living in private households (*N* = 22,072; 59% response rate). Inequality indicators based on the PROGRESS-Plus framework were used to disentangle existing social intersections. To identify intersectional groups, decision tree models, including classification and regression trees (CARTs), chi-squared automatic interaction detector (CHAID), conditional inference rees (CITs), and C5.0, along with an ensemble algorithm, extreme gradient boosting (XGBoost), were applied.

**Results:**

XGBoost (AUC 78.8%) identified regional differences (Autonomous Community) as the most important factor for classifying BCS attendance, followed by education, age, and marital status. The C5.0 model (balanced accuracy 81.1%) highlighted that the relative importance of individual characteristics, such as education, marital status, or age, for attendance differs based on women’s place of residence and their degree of interaction. The highest risk of not attending BCS was observed among illiterate older women in lower social classes who were born in Spain, were residing in Asturias, Cantabria, Basque Country, Castile and León, Extremadura, Galicia, Madrid, Murcia, La Rioja, or Valencian Community, and were married, divorced, or widowed. Subsequently, the risk of not attending BCS extends to three other groups of women: women living in Ceuta and Melilla; single or legally separated women living in the rest of Spain; and women not born in Spain who were married, divorced, or widowed and not residing in Ceuta or Melilla.

**Conclusion:**

The combined use of decision trees and ensemble algorithms can be a valuable tool in identifying intersectional positions at a higher risk of not utilizing public resources and, thus, can aid substantially in developing targeted interventions to increase BCS attendance.

## 1 Introduction

In the majority of EU countries, universal health insurance and, in theory, universal access to healthcare are in place. However, while many have achieved universal health coverage for a broad range of services, access to these services is not equally experienced by all citizens (1).

The Conceptual Framework of Access to Healthcare developed by Levesque et al. presents a comprehensive multidimensional approach to understanding and improving health service accessibility that combines individual- and system-related variables (2). In this article, we focus on an individual-level accessibility outcome, such as attendance at organized breast cancer screening programs, and examine groups at the intersections of individual-, system-, and environment-level inequality dimensions to identify those at the highest risk for non-attendance.

In 2020, breast cancer was the most diagnosed cancer among women, with 2.3 million cases, and the most prevalent, with 7.8 million women still living after a diagnosis within the past 5 years, globally (3). According to the European Cancer Information System (ECIS), breast cancer accounted for 28.7% of all cancers in women in the EU-27 countries in 2020, with an estimated 355,457 new cases and an age-standardized mortality rate (ASR) of 142.8 per 100,000 inhabitants (4). In Spain, the context for this study, breast cancer constitutes 30.7% of all cancers in women, with 110,946 new cases detected in 2020. The ASR for breast cancer is 132 per 100,000, followed by colorectal cancer with an ASR of 58.4 per 100,000 (4).

The importance of early breast cancer detection has been on the European agenda since 1980. The European Commission has urged Member States to establish preventive organized screening programs (OSPs) that bi-annually invite women aged 50–69 years for breast cancer screening (BCS). Additionally, women aged 45–49 years and 70–74 years should be conditionally screened every 2–3 years and 3 years, respectively (5). Over time, Member States have implemented OSPs with age ranges ranging from 40 to 74 years (6).

In 1990, Spain established OSPs, making it one of the first European countries to do so. However, due to the fact that some competencies of the Spanish healthcare system are allocated at the level of Autonomous Communities (regions), there were substantial differences between regions in invitation schedules: Some regions implemented a bi-annual regimen for women aged 45–69, some only for age groups 50–69, and there were differences with regards to the starting points as well—for example, Navarre was the first region implementing the program in 1990, followed by Catalonia, Castilla-La Mancha, Castile and León, Catalonia, Valencian Community, and Galicia in 1992. Ceuta and Melilla, on the other hand, only started their program in 2005 (7). The national attendance rate has steadily increased over the years, with 72.6% of targeted women attending their last medical appointment and 93.13% undergoing screening at least once in their lifetime in 2020. (8). Although this is a small proportion (6.87%), there is a large number of invited women who did not seek care, despite having knowledge of their age-related risks and available healthcare services.

Studies have shown that attendance inequality exists in BCS, with non-attendance not being uniformly distributed among the targeted population. In a recent international systematic review, Mottram et al. found that migrant women, women of lower socioeconomic status, non-homeowners, and those who previously experienced false-positive results had the lowest attendance rate (9). Recent studies in Spain have found that being married, having Spanish nationality, and having higher education are positive predictors of screening participation (10). Similarly, Serral et al. stated that socioeconomic status and country of origin are the strongest discriminating factors for screening attendance in Spain (11). Moreover, Martín-López et al. reported that holding Spanish nationality, being married, and having a high educational and income level are positively associated with BCS attendance (12). However, so far and to the authors’ knowledge, no study has assessed these sociodemographic variables alongside the regional importance of non-attendance rates. In decentralized healthcare systems such as the Spanish system, where the concentration of health professionals in certain autonomous regions is approximately twice that of others, it is crucial to assess the influence of regional disparities on healthcare accessibility (13, 14).

Moreover, previous studies have examined dimensions of inequality as independent predictors of non-attendance. However, adopting an *intersectional* framework is necessary to understand the complexity of attending breast cancer screening by analyzing social and demographic risk factors beyond independent predictors.

The theory of intersectionality, developed in 1991 by Kimberlé Crenshaw in the context of unfair legal processes for Black women, posits that discrimination and oppression result from the intersection of multiple aspects of identity (e.g., gender, ethnicity) and related experiences (15). This theoretical framework is used in several disciplines today, with a broader range of variables that embody social power structures (e.g., sexuality, age, and income) (16). Inequalities in health-related outcomes, such as screening behavior, can be disentangled by adopting a non-additive, intersecting approach (17). In other words, experiences of discrimination by individuals who lay at the intersection of several axes of inequalities cannot be assessed and understood only by summing up the oppression suffered by these two variables independently.

Although traditionally examined in qualitative studies, recent years have witnessed a significant increase in the use of quantitative methods for intersectionality research (18). In this study, relatively simple regression analyses with interaction terms or intersection variables are more frequently used; more complex and less frequently used approaches include analyses such as decomposition analysis or decision trees (18). The latter refers to machine learning techniques that allow a non-parametric, data-driven exploration of intersectional heterogeneity in the data. In this study, variables potentially discriminating between groups varying in their estimated prevalence of a health outcome, such as PROGRESS-Plus characteristics, can be included in the analysis without the risk of overfitting the model (19). Even at the risk of non-replicative arbitrary data splits, decision trees allow for any interaction, which helps, on the one hand, with variable selection and reduction and, on the other hand, identifying subgroups for potential interventions (20, 21).

Only a few public health studies have used decision trees to explore sociodemographic inequalities. Mena et al. used classification and regression trees (CARTs) and conditional inference trees (CITs) to identify groups with a higher prevalence of non-daily vegetable intake (22). Eagle et al. employed chi-squared automatic interaction detection trees (CHAID) to explore the associations between race and ethnicity, sex, depression, and concussion history with reported suicide attempts among adolescents in the US (23). Delgado-Gallegos et al. used C5.0 to assess perceived stress in healthcare professionals attending to COVID-19 cases in Mexico (24). These studies suggest that decision trees are a viable and helpful tool to identify the intersectional groups at high risk for specific outcomes, but so far, only a few studies have employed decision trees for studying sociodemographic inequalities in attending breast cancer screening.

Freitas et al. used CHAID to examine individual and environmental factors that predict breast cancer screening practices among women aged 45–69 in Portugal (25), but have not adopted a decidedly intersectional perspective. To the author’s knowledge, no study in Spain has addressed inequality in breast cancer screening from an intersectional perspective or using machine learning techniques. Thus, according to national and regional breast cancer screening guidelines and based on the intersections of PROGRESS-Plus characteristics, this study aims to identify the intersectional positions of women more at risk of not undergoing breast cancer screening in Spain.

## 2 Materials and methods

### 2.1 European health interview survey

We used the cross-sectional data from the third wave of the European Health Interview Survey (EHIS) conducted in Spain in 2019 and 2020 to identify intersectional groups. The sample size of the survey was 22,072 respondents, corresponding to 59% of the invited participants (*N* = 22,072; 59% response rate). The survey, legally binding from the second wave onwards, is conducted every 5 years (every 6 years since 2019) and targets the population over 15 years old living in private households. Its main objective is to gather data on the population’s health status, the utilization of health services, and determinants of health in a harmonized and comparable manner at the European level (b). The survey is framed within the European Commission’s (EC) Regulation 1338/2008, and the current legally binding EC Regulation describing the survey’s definitions is EC 2018/255 of 19 February 2018 (26, 27).

### 2.2 Ethical consideration

No ethical approval is required for this study as it is a secondary analysis of de-identified publicly available data.

### 2.3 Primary outcome

The primary outcome of this study was self-reported breast cancer screening attendance at some point during their lifetime via mammography for women aged 45–69 in 7 out of 19 Autonomous Communities (Castilla-La Mancha, Castile and León, Valencian Community, La Rioja, Navarre, Ceuta, and Melilla) and for women aged 50–69 in the rest of Autonomous Communities. Possible answers to these questions were: yes, no, unknown, or unanswered. Answers were dichotomized, removing those few who responded unknown or unanswered to avoid incorporating uncertainty on whether the respondent reported never attending BCS (no = 0, yes = 1).

### 2.4 Explanatory variables

The explanatory variables used in this research are based on the PROGRESS-Plus framework (28). Their use to disentangle social inequalities has been extensively discussed (29) and used (30). For this study, we categorized existing indicators of the survey into the PROGRESS-Plus categories and used them as potential predictors of non-attendance at BCS.

*Place of Residence* was determined through two different variables: the size of the municipality and the specific Autonomous Community. The first variable was composed of the following seven categories: less than 10,000 inhabitants, 10,000–20,000 inhabitants, 20,000–50,000 inhabitants, 50,000–100,000 inhabitants, more than 100,000 inhabitants, capital of the province, or more than 500,000 inhabitants. As mentioned above, the specific Autonomous Community also serves as a proxy for the time period of the implementation of BCS programs and the density of health professionals in the region.

*Race, ethnicity, culture, and language* were measured by the respondents’ country of origin. The information provided by this variable in the *EHIS* was whether the participants were born in Spain or another country. Thus, the variable was employed as a binary variable (Spain = 1 and other = 2).

*Occupation* was operationalized through the respondents’ current working situation. The derived categorical variable had the following categories: paid employment, unemployed, retired, unable to work, (unpaid) household work, and others. The “others” category is a merged category that includes *EHIS* classification such as unable to work, studying, and others, as the two first categories had very few participants, 17 and 1, respectively.

*Sex* was not included as an explanatory variable but rather as a requirement needed for including participants in the analysis: to be a female. *Gender* and *religion* dimensions were not included in the present research, as the *EHIS* did not comprise any questions covering gender identification or religious preferences.

*Education* was measured following the CNED14 classification, the Spanish adaptation of ISCED-2011 (31). The variables employed had the following categories: illiterate, uncompleted primary education, completed primary education, first-stage high school, finished high school, intermediate vocational training, superior vocational training, and university degree.

*Socioeconomic status* was operationalized by the type of occupation. The variable’s classification was obtained based on the Spanish Society of Epidemiology classification developed in 1995 and revised in 2012. This grouping comprises 6 groups, with 1 being the highest social class and 6 being the lowest (32).

*Social capital* was not explicitly included in the dataset; however, we used two proxy variables: marital status (single, married, legally separated/divorced, or widowed; indicating the availability of spousal support) and type of household (alone, with a partner, with a partner and children, alone with children, or others; indicating the availability of family support).

The *Plus* dimension of the PROGRESS-Plus framework was proposed to extend the social determinants of health that could potentially be discriminating features. Among these contextually dependent factors, characteristics that attract discrimination, features of relationships, and time-dependent relationships have been highlighted (33). In this study, we assessed characteristics that attract discrimination through the Global Activity Limitations Indicator (GALI) and age (34). The GALI self-reported measure is conceived by assessing the degree of limitation experienced in the last 6 months, with the possible answers being severely limited, mildly limited, or not limited. Age is a categorical variable comprising women aged 45–69 in quintiles (45–49 years old, 50–54 years old, 55–59 years old, 60–54 years old, and 65–69 years old). While one would anticipate a proportional relationship between age and BCS attendance (i.e., older women being invited to BCS more times in their lives than younger women), this variable was included to assess its potential interactions with other PROGRESS-Plus variables. Specifically, it aimed to examine whether distinct social determinants of health intersect in different age groups.

As a final point, none of the categories of the explanatory variables were collapsed (e.g., illiterate and uncompleted primary education); Instead, they were included in the model individually with all categories. This approach was chosen because decision tree algorithms permit a high level of granularity and the ability to capture interactions and potential intersections among the various categories (35).

### 2.5 Analytic approach

Descriptive analytics, frequencies, and percentages were calculated for all variables. The statistical significance between participants who attended BCS and those who did not was tested using a chi-squared test for all variables.

For this study, a complete case analysis was conducted when missing data were present. The total sample size was restricted to women aged 45–69 in 7 out of 19 Autonomous Communities (Castilla-La Mancha, Castile and León, Valencian Community, La Rioja, Navarre, Ceuta, and Melilla) and to 50–69 years in the rest of Autonomous Communities (*n* = 4,180). Among these women, those who did not provide information on whether they ever underwent mammography (*n* = 8) were excluded, and those who did not provide their marital status (*n* = 21) were excluded. Hence, the final total sample size of the study was 4,151 participants.

We used decision trees to identify the groups of women most at risk of not attending BCS. There is scarce literature that suggests which decision trees better operate on diverse health outcomes. CART bases its splitting decision on the lowest Gini impurity (or entropy) coefficient of every possible split (21). It is often criticized for not providing statistical significance measures and being biased toward variables with many categories. CIT was developed to overcome some of CART’s limitations. CIT employs a formal statistical hypothesis for building the decision trees and avoids variable selection bias by dividing the selection process into two steps (36). CHAID was the first decision tree developed based on statistical significance tests, in which all covariates are tested against the outcome, and the one with the highest association is selected as the splitting variable. The limitation of CHAID is that it computes only categorical variables and is based on the chi-squared test (37). C5.0 is a non-parametric method that uses the entropy of the imputed variables to generate splits. Therefore, nodes are generated based on the data split that produces a higher information gain (i.e., the entropy of the data split is the lowest, meaning the homogeneity among the included cases is the highest) and therefore appears to be more advantageous for classification (38). After C5.0 generates a vast tree, it applies the binomial confidence limit method to every subtree for pruning through a high predicted error rate. Moreover, C5.0 uses adaptive boosting (39) and winnowing in the growing process (40, 41).

In the present study, the four most commonly used decision trees were implemented using R packages “caret” (42) for exploratively hypertuning the models and “rpart” (CART) (43), “chaid” (CHAID) (44), “partykit” (CIT) (20), and “C50” (C5.0) (40) for outputting the final model in R version 4.2.3.

Decision trees that use classification algorithms employ several metrics derived from a confusion matrix to evaluate their performance. In the case of a binary outcome, the confusion matrix is a 2 × 2 table that illustrates the total number of true positives (TP – hit), true negatives (TN – correct rejection), false negatives (FN – type II error), and false positives (FP – type I error) when comparing the predicted positive (PP) and predicted negative (PN) cases to the actual positive (P) and actual negative (N) cases. Measures such as balanced accuracy, recall, precision, and F1 score (Table 1) can then be calculated and used for model performance evaluation (45).

**Table 1:** Informative metrics derived from the confusion matrix for model’s performance evaluation.

Evaluation metrics	Equation
Balanced accuracy	$\frac{\frac{TP}{P}+\frac{TN}{N}}{2}$
Recall (sensitivity)	$\frac{TP}{P}$
Precision	$\frac{TP}{PP}$
F1 score	$\frac{2\;TP}{2\;TP+FP+FN}$

Despite decision trees being reliable tools for intersectional subgroup identification, they are unstable given their dependence on the data used to train them and, thus, posing the risk of overfitting. Small changes in the training data could lead to differences in decision tree construction; hence, the implementation of ensemble algorithms is recommended (46). There are several ensemble algorithms that employ different procedures, such as bagging (Random Forest), boosting (AdaBoost), and bagging and boosting (extreme gradient boosting). Following the results of previous research (47–49), we decided to use extreme gradient boosting (XGBoost) to increase the internal validation and robustness of the decision trees. The algorithm was implemented using the package “xgboost” (49) in R version 4.2.3.

## 3 Results

### 3.1 Descriptive statistics of the sample

A summary of the descriptive statistics of the sample can be found in Table 2. Association chi-squared tests suggest marginal associations between each independent variable and the outcome, except for municipality size. The total sample size is 4,151. Of those, 3,886 attended BCS during the last 2 years, while 285 did not. As expected, the youngest group, women aged 45–49 years, attended less in their lifetime (18.34% – never attended) than older women (*χ*^2^ = 76.93, df = 4, *p* < 0.001). Women not born in Spain (14.78%) attended less BCS compared to those born in Spain (*χ*^2^ = 35.54, df = 1, *p* < 0.001). Illiterate women (43.75%) (*χ*^2^ = 74.94, df = 7, *p* < 0.001) and women in the lowest social class (10.51%) (*χ*^2^ = 38.00, df = 6, *p* < 0.001) had the smallest number of attendances at BCS. Regarding the place of residence, women living in municipalities with 10,000–20,000 inhabitants (8.82%) (*χ*^2^ = 16.95, df = 6, *p* < 0.151) and women living in Melilla (37.14%) (*χ*^2^ = 157.26, df = 18, *p* < 0.001) had the lowest attendance at BCS. Finally, legally separated women (13.39%) (*χ*^2^ = 68.11, df = 4, *p* < 0.001), women living alone with children (8.21%) (*χ*^2^ = 27.8, df = 4, *p* < 0.001), women in a working situation labeled as other (10.90%) (*χ*^2^ = 26.92, df = 4, *p* < 0.001), and severely limited women (12.44%) (*χ*^2^ = 13.15, df = 2, *p* < 0.0106) participated in BCS the least among their PROGRESS-Plus dimension.

**Table 2:** Descriptive PROGRESS-Plus characteristics on the attendance to BCS among targeted women in Spain based on the 2020 EHIS.

	Attended BCS (*N* = 3,866)	Never attended BCS (*N* = 285)	Total (*N* = 4,151)	*p*-value
**Age quintiles (years)**				
45–49	187 (4.8%)	42 (14.7%)	229 (5.5%)	<0.001
50–54	865 (22.4%)	93 (32.6%)	958 (23.1%)	
55–59	982 (25.4%)	63 (22.1%)	1,045 (25.2%)	
60–64	956 (24.7%)	48 (16.8%)	1,004 (24.2%)	
65–69	876 (22.7%)	39 (13.7%)	915 (22.0%)	
Country of origin				
Spain	3,572 (92.4%)	234 (82.1%)	3,806 (91.7%)	<0.001
Other	294 (7.6%)	51 (17.9%)	345 (8.3%)	
**Educational group**				
Illiterate	19 (0.5%)	13 (4.6%)	32 (0.8%)	<0.001
Uncompleted primary education	186 (4.8%)	23 (8.1%)	209 (5.0%)	
Completed primary education	720 (18.6%)	63 (22.1%)	783 (18.9%)	
First-stage high school	976 (25.2%)	75 (26.3%)	1,051 (25.3%)	
Completed high school	533 (13.8%)	30 (10.5%)	563 (13.6%)	
Intermediate vocational training	331 (8.6%)	18 (6.3%)	349 (8.4%)	
Superior vocational training	266 (6.9%)	22 (7.7%)	288 (6.9%)	
University degree	835 (21.6%)	41 (14.4%)	876 (21.1%)	
**Socioeconomic position**				
1 (high)	427 (11.0%)	17 (6.0%)	444 (10.7%)	<0.001
2	333 (8.6%)	14 (4.9%)	347 (8.4%)	
3	851 (22.0%)	47 (16.5%)	898 (21.6%)	
4	420 (10.9%)	21 (7.4%)	441 (10.6%)	
5	1,104 (28.6%)	101 (35.4%)	1,205 (29.0%)	
6 (low)	545 (14.1%)	64 (22.5%)	609 (14.7%)	
Not classifiable	186 (4.8%)	21 (7.4%)	207 (5.0%)	
**Size of the municipality (people)**				
<10,000	484 (12.5%)	34 (11.9%)	518 (12.5%)	0.151
10,000–20,000	920 (23.8%)	89 (31.2%)	1,009 (24.3%)	
20,000–50,000	314 (8.1%)	15 (5.3%)	329 (7.9%)	
50,000–100,000	350 (9.1%)	19 (6.7%)	369 (8.9%)	
>100,000	590 (15.3%)	56 (19.6%)	646 (15.6%)	
Province Capital	436 (11.3%)	25 (8.8%)	461 (11.1%)	
>500,000	772 (20.0%)	47 (16.5%)	819 (19.7%)	
**Autonomous community**				
Andalusia	443 (11.5%)	52 (18.2%)	495 (11.9%)	<0.001
Aragon	154 (4.0%)	2 (0.7%)	156 (3.8%)	
Asturias	176 (4.6%)	13 (4.6%)	189 (4.6%)	
Balearic Islands	61 (1.6%)	8 (2.8%)	69 (1.7%)	
Canary Islands	212 (5.5%)	12 (4.2%)	224 (5.4%)	
Cantabria	165 (4.3%)	13 (4.6%)	178 (4.3%)	
Castile and León	188 (4.9%)	14 (4.9%)	202 (4.9%)	
Castilla-La Mancha	228 (5.9%)	19 (6.7%)	247 (6.0%)	
Catalonia	356 (9.2%)	7 (2.5%)	363 (8.7%)	
Valencian Community	393 (10.2%)	28 (9.8%)	421 (10.1%)	
Extremadura	149 (3.9%)	8 (2.8%)	157 (3.8%)	
Galicia	217 (5.6%)	15 (5.3%)	232 (5.6%)	
Madrid	384 (9.9%)	20 (7.0%)	404 (9.7%)	
Murcia	144 (3.7%)	9 (3.2%)	153 (3.7%)	
Navarre	170 (4.4%)	7 (2.5%)	177 (4.3%)	
Basque Country	192 (5.0%)	12 (4.2%)	204 (4.9%)	
La Rioja	140 (3.6%)	8 (2.8%)	148 (3.6%)	
Ceuta	50 (1.3%)	12 (4.2%)	62 (1.5%)	
Melilla	44 (1.1%)	26 (9.1%)	70 (1.7%)	
**Marital status**				
Single	548 (14.2%)	83 (29.1%)	631 (15.2%)	<0.001
Married	2,269 (58.7%)	122 (42.8%)	2,391 (57.6%)	
Widowed	460 (11.9%)	27 (9.5%)	487 (11.7%)	
Legally separated	194 (5.0%)	30 (10.5%)	224 (5.4%)	
Divorced	395 (10.2%)	23 (8.1%)	418 (10.1%)	
**Type of household**				
Alone	1,024 (26.5%)	87 (30.5%)	1,111 (26.8%)	<0.001
With partner	1,130 (29.2%)	49 (17.2%)	1,179 (28.4%)	
With a partner and children	918 (23.7%)	66 (23.2%)	984 (23.7%)	
Alone with children	556 (14.4%)	65 (22.8%)	621 (15.0%)	
Other	238 (6.2%)	18 (6.3%)	256 (6.2%)	
**Working situation**				
In paid employment	1742 (45.1%)	129 (45.3%)	1,871 (45.1%)	<0.001
Unemployed	355 (9.2%)	37 (13.0%)	392 (9.4%)	
Retired	903 (23.4%)	34 (11.9%)	937 (22.6%)	
Household work (unpaid)	727 (18.8%)	68 (23.9%)	795 (19.2%)	
Others	139 (3.6%)	17 (6.0%)	156 (3.8%)	
**Experienced limitation**				
Severely limited	176 (4.6%)	25 (8.8%)	201 (4.8%)	0.0106
Mildly limited	960 (24.8%)	55 (19.3%)	1,015 (24.5%)	
Not limited	2,730 (70.6%)	205 (71.9%)	2,935 (70.7%)	

* The outcome is a binarized variable that measures whether the respondent never attended breast cancer screening (yes = 0, no = 1).

### 3.2 Data analysis: pre-processing steps

Several pre-processing steps were taken to prepare the data to be analyzed. First, a complete case analysis was conducted, as only 29 cases had missing data on any variable. Second, to improve external validity, data were randomly split into a training subset (*N* = 3,321, 80% of the entire dataset) and a testing subset (*N* = 830, 20% of the entire dataset), with stratification of the outcome (i.e., to respect the distribution of classes in both subsets). Furthermore, balance sampling methods were applied to the training data given the imbalanced nature of the dependent variable (93.13% ever attended BCS, 6.87% never attended). Several studies have demonstrated that model performance improves when resampling the training data in highly imbalanced datasets (50–52). Four balanced sampling techniques that minimize the majority class (undersampling), maximize the minority class (oversampling), or minimize the majority class and maximize the minority class (SMOTE and ROSE) were tested (Table 3). Oversampling outperformed the other sampling techniques, estimated through the area under the curve (AUC) of logistic regression (LR).

**Table 3:** Performance of balance sampling techniques on the training dataset.

Balancing technique	Never attended BCS	Attended BCS	Total	AUC (LR)
Oversampling	3,101	3,087	6,188	0.714
Undersampling	234	236	470	0.691
ROSE sampling	1,677	1,644	3,321	0.701
SMOTE sampling	3,087	3,087	6,174	0.708

### 3.3 Data analysis: explanation of the tree

**Figure 1:**
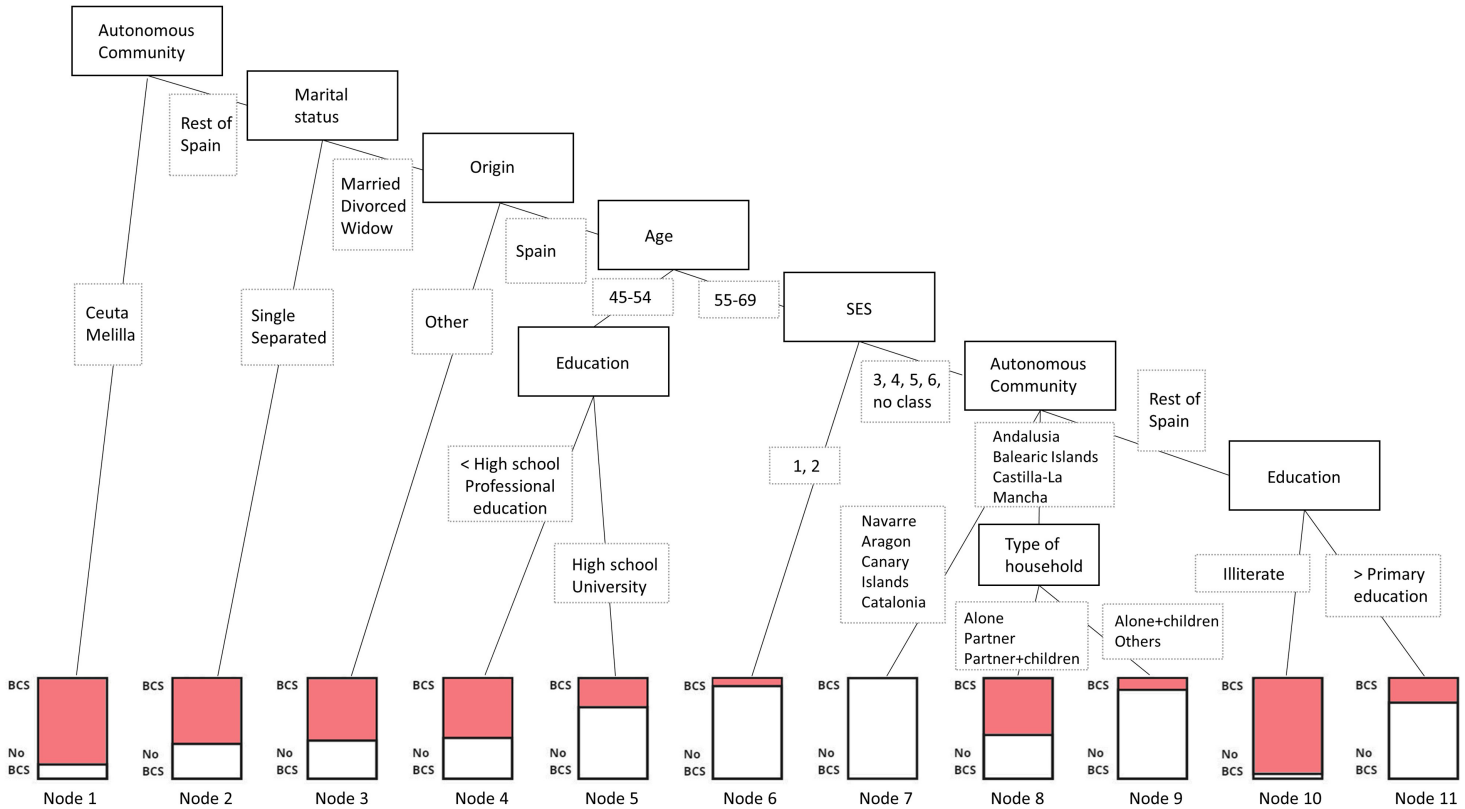
C5.0 decision tree assessing intersectional social positions most at risk of not attending BCS.

We tested several decision trees, CART, CIT, CHAID, and C5.0, to the oversampled training data. C5.0 outperformed the others with a balanced accuracy of 81.1%, recall of 80.7%, precision of 24.3%, and F1 score of 0.374. See Supplementary material 1 for more details and Figure 1; Table 4 for the final C5.0 decision tree.

**Table 4:** Description of the intersectional social positions (nodes) at risk of not attending BCS from the C5.0 decision tree.

Rank	Intersectional social positions	Node number
1	Illiterate women living in Asturias, Cantabria, Castile and León, Valencian Community, Extremadura, Galicia, Madrid, Murcia Basque Country or La Rioja, from middle and low social classes, aged 55 or older, born in Spain, married, divorced, or widowed	10
2	Women living in Ceuta or Melilla	1
3	Single or separated women not living in Ceuta or Melilla	2
4	Married, divorced, or widowed women born outside Spain and not living in Ceuta or Melilla	3
5	Married, divorced, or widowed women, born outside Spain and not living in Ceuta or Melilla, younger than 55 years of age with less than high school education or professional education	4
6	Married, divorced, or widowed women, living alone, with a partner, or with a partner and children, born outside Spain, older than 55 years of age, belonging to social classes 3–6, living in the Autonomous Communities of Andalusia, Balearic Islands, or Castilla-La Mancha	8
7	Married, divorced, or widowed women, born outside Spain and not living in Ceuta or Melilla, younger than 55 years of age with more than high school education (except professional education)	5
8	Women living in Asturias, Cantabria, Castile and León, Valencian Community, Extremadura, Galicia, Madrid, Murcia Basque Country or La Rioja, from middle and low social classes, with primary education or greater, aged 55 years or older, born in Spain, married, divorced, or widowed	11
9	Married, divorced, or widowed women, living alone with children or other household constellations, born outside Spain, older than 55 years of age, belonging to social classes 3–6, living in the Autonomous Communities of Andalusia, Balearic Islands, or Castilla-La Mancha	9
10	Married, divorced, or widowed women, born outside Spain and not living in Ceuta or Melilla, older than 55 years of age, belonging to the highest two social class groups	6
11	Married, divorced, or widowed women born outside Spain, older than 55 years of age, belonging to social classes 3–6, living in the Autonomous Communities of Navarre, Aragon, Canary Islands, or Catalonia	7

The best tree identified the variables Autonomous Communities, origin, marital status, age, education, socioeconomic class, origin, and type of household as relevant in explaining attendance and non-attendance to BCS. The first splitting point, the root node, is found in Autonomous Communities, where women living in Ceuta or Melilla form a subgroup (Node 1, 464 cases /71 no-cases), and the rest of Spain further split. The second split is found in marital status, where single or separated women create a subgroup (Node 2, 1,112/585), and married, divorced, and widowed women further split. The third node relates to the respondent’s origin country: women born outside Spain form a subgroup (Node 3, 280/163), and women born in Spain proceed to split. Women born in Spain, married, divorced, or widowed, and not residing in Ceuta or Melilla are additionally divided based on being 55 years of age or older. The youngest group is further split based on the level of education, forming two final leaves: less than high school education or professional education (Node 4, 496/334) and greater than high school education except for professional education (Node 5, 93/221). The oldest group is fragmented based on the socioeconomic class of the respondents, where those belonging to the highest two social classes form a subgroup (Node 6, 30/312), and the rest is additionally divided based on the place of residence. The sixth split divides the abovementioned group into those living in the Autonomous Communities of Navarre, Aragon, Canary Islands, or Catalonia (Node 7, 0/316), those living in Andalusia, Balearic Islands, or Castilla-La Mancha, and the rest of Spain. Women living in Andalusia, Balearic Islands, or Castilla-La Mancha are further separated based on the type of household they live in: alone, with a partner, or with a partner and children (Node 8, 307/243), and those living alone with children or other household constellations (Node 9, 7/48). Finally, women living in Asturias, Cantabria, Castile and León, Valencian Community, Extremadura, Galicia, Madrid, Murcia, Basque Country, or La Rioja, from middle and low social classes, aged 55 years or older, born in Spain, and married, divorced, or widowed, are lastly split based on their level of education: illiterate (Node 10, 42/2) and primary education or greater (Node 11, 270/792).

**Figure 2:**
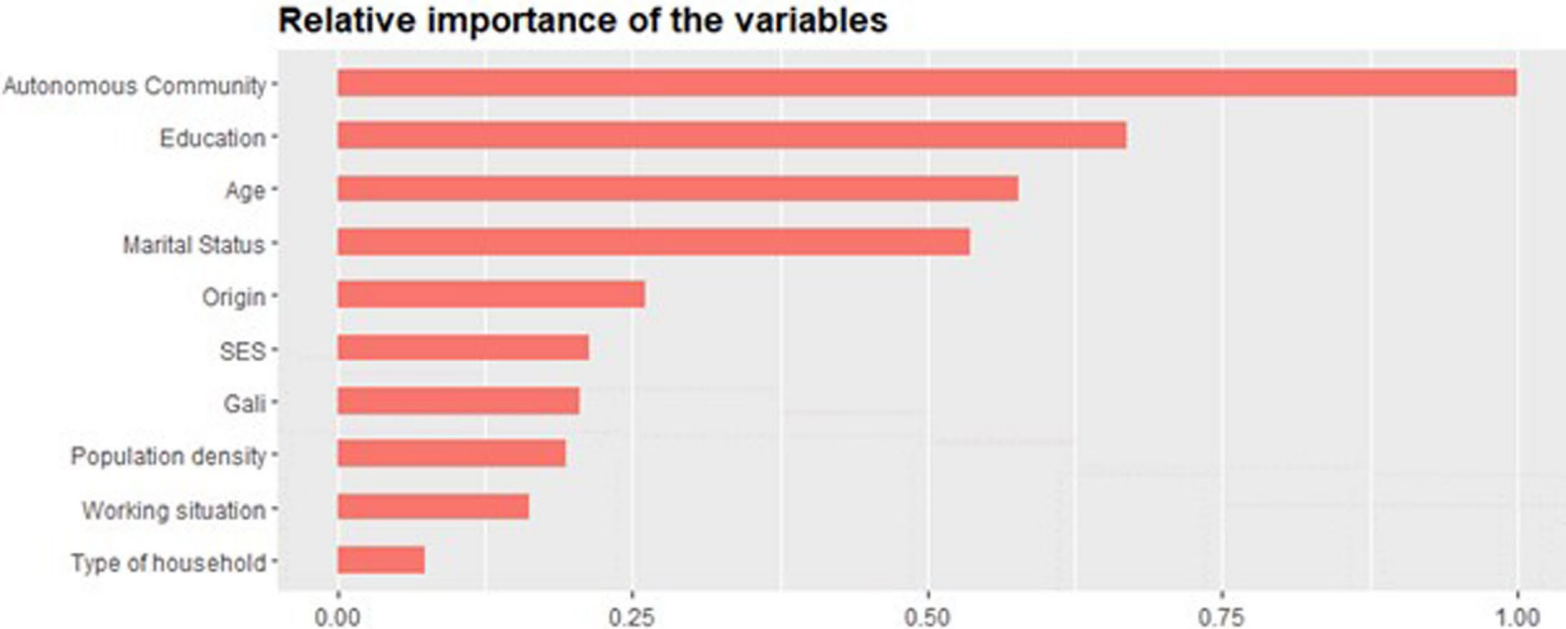
Variables’ relative importance in the XGBoost model for predicting BCS attendance.

For more robust information and internal validation of the decision tree, extreme gradient boosting was performed. XGBoost sequentially trains multiple decision trees (bagging), where each tree endeavors to correct the classification errors of the precious one by assigning specific weights to each tree and its leaves (boosting) (49). The parameters used to build the ensemble algorithm were a learning rate of 0.1 (low learning rate to be more robust to overfitting), a maximum number of boosting iterations of 53, and a learning objective of logistic regression for binary classification. XGBoost assessed the importance of the variables when building the boosted trees through the information gain criteria. In essence, this algorithm informs which variables were more significant in constructing the decision trees and therefore has a higher predictive power in the model. With an AUC of 78.80%, the algorithm resolved that Autonomous Community followed by education, age, and marital status are the most important variables for predicting whether targeted women in Spain will attend BCS or not. Following with less than 50% relative importance are origin, social class, GALI, population density, working situation, and type of household (Figure 2).

## 4 Discussion

In this study, we employed decision trees to identify intersectional groups of women at the highest risk for never attending breast cancer screening and ensemble algorithms to give more robustness to the classification model. Autonomous Community, education, age, and marital status, were reported as the most critical variables for classifying attendance at BCS among women aged 45–69 in Spain. While all Autonomous Communities have now adopted the OSP, it is worth noting that Ceuta and Melilla were the last to implement in 2006, and these were the regions where the lowest number of targeted women attended screenings (53). In addition, these regions are those with the lowest density of health professionals (2.5/1,000 inhabitants) in Spain (14). Moreover, given the enclave of these cities in Morocco, many inhabitants define themselves as Muslim, and several studies have observed that Muslim women have a lower attendance at BCS (54). Finally, the average level of education of women in these regions is significantly lower than in the rest of Spain (8).

Autonomous Communities, such as Navarre (1990) and Catalonia (1992), where OSPs were first implemented sustain higher screening attendance (7, 53). Indeed, our analysis enhances the importance of the intersections of individual and system levels of inequality. While women in higher social classes tend to attend BCS more than those in low and middle social classes (9), our analysis shows that women born in Spain, married, divorced, or widowed, not living in Ceuta or Melilla from the two highest social classes, have a slightly higher risk of not attending BCS compared to women born in Spain, married, divorced, or widowed, with middle and low social class, living specifically in Navarre, Aragon, Canary Islands, and Catalonia. These Spanish regions are thus strongly protective factors for BCS attendance, potentially over individual-level variables such as social class.

Among women born in Spain, married, divorced, or widowed, not living in Ceuta or Melilla, and younger than 55 years of age, their education level determines their risk of not attending BCS. Women with lower education have been at higher risk of not attending BCS compared to those with higher education (12, 55). However, among women older than 55 years of age, social class is the determinant variable. In this case, those in the two highest social classes are a subgroup with the second lowest predicted risk of not attending BCS. The non-linear nature of the interactions permitted in decision trees allows for the emergence of such divergences.

Another unexpected intersectional result of this analysis is the intersection between marital status and origin. Women not living in Ceuta and Melilla, single or separated, and who are married, divorced, or widowed with a migration background have a similarly high risk of not attending BCS. Single and separated women have a higher risk of not attending BCS than women with migration backgrounds who are married, divorced, or widowed. In other words, in this study, the origin of women plays a decisive role only for those who are not single or separated. Although contradictory to previous studies (11, 56, 57), marital status plays a more prominent role than migration background. These results are reinforced by the variable importance rank for building the model, where marital status has double the importance of origin.

Finally, the intersectional subgroup with the highest risk of not attending BCS, Node 10, encompassed women with very low educational levels in the Autonomous Communities of Asturias, Cantabria, Basque Country, Castile and León, Extremadura, Galicia, Madrid, Murcia, La Rioja, and Valencian Community. Although this subgroup intersects with the privileged position of other variables, such as marital status, age, and origin, being illiterate strongly determines their risk of not attending BCS. These results align with an earlier study showing that education is essential for BCS attendance (55). The present study contributes to and strengthens this statement showing that, although lying at the intersection of several protective factors, intersecting with low levels of education is pernicious. Furthermore, such social inequalities will persist if the responsible public health institutions do not address them. Molina-Barceló et al. found that most of the Autonomous Communities of this subgroup, Asturias, Castile and León, Extremadura, Madrid, and Murcia, did not develop interventions to decrease inequalities in the uptake of BCS in the last decade (58).

Although our results are specific to the Spanish healthcare system, some aspects might have implications beyond Spain. When healthcare competencies such as the allocation of financial resources, organization of territorial coverage, or development and implementation of preventive programs are delegated to regional authorities, disparities in access to healthcare services may emerge (13). This study identified the Autonomous Community as the most pivotal variable for predicting whether a woman aged 45–69 years old in Spain will attend BCS. Indeed, the Spanish Federation of Breast Cancer has, for several years, been advocating for greater interregional political cohesion to enhance equitable access to breast cancer prevention and treatment (59). Regions with lower economic means, such as Ceuta and Melilla, have invested less in addressing social inequalities compared to wealthier regions, such as Navarre or Catalonia, ultimately enlarging inequities within and between regions (58). Regional differences in the use of health services and their correlation to the socioeconomic level of the regions have been previously found in Spain (60). While a trend toward narrowing this gap has been observed, Autonomous Communities in the south and northwest of the country continue to exhibit worse health indicators than those in the north and northeast. This is particularly notable in regions such as Ceuta, Melilla, Canary Islands, and Andalusia (61).

### 4.1 Strengths and limitations

Supervised machine learning techniques, specifically decision trees and the presented ensemble algorithm, allow for a non-parametric and non-linear explanation of the relationship between explanatory variables and the outcome. Thus, unlike the classic statistical analysis, where including many potential predictors could lead to overfitting the model, machine learning techniques allow for a more comprehensive inclusion of all available PROGRESS-Plus variables in the dataset. Multicollinearity deriving from potentially correlated predictors is not an issue in machine learning approaches since decision trees split based on an individual variable at each node, and ensemble algorithms further mitigate the risk by outputting the average importance of each variable. In this study, machine learning algorithms allowed an inductive exploration of intersecting social determinants of health in women. At last, no predictive value is tested; instead, variables are evaluated as potential predictors for building the classification models (35).

The study is not without limitations. No causal inference can be drawn due to the cross-sectional design of the survey. Moreover, response and common-method bias may occur due to the nature of the self-reported survey. In fact, the response rate of *EHiS* wave 3 was 59%, emphasizing the need for caution when drawing conclusions from studies using this dataset. Previous researchers have speculated that the *EHiS* suffers from underestimating inequalities in access to screening programs (58). Along these lines, the nature of the target population in the survey—people living in private households—could potentially reveal details about individuals living in less advantaged housing situations, such as public or institutional housing. Furthermore, although the presented decision tree outperformed others, it is relatively large and outputs both simple and highly dimensional intersectional positions. Therefore, the large dimensionality of some subgroups might challenge the usability of their content. Indeed, the present analysis ought to enhance the understanding of the intersectional importance of different variables and describe those most at risk of not attending the BCS. Finding the balance between algorithm performance and public health interpretability and usability is essential in health prevention.

Finally, secondary data intrinsically limit the included variables and their categorization. The dimension of race/ethnicity could only be estimated based on the respondent’s country of origin, which does not fully capture the experience of discrimination that women might experience. An extensive body of literature shows how race/ethnicity is often a proxy for convergence factors such as experiences of discrimination, social mobility, and a lack of financial opportunities (62).

## 5 Conclusion

The present study pinpointed the intersectional positions of women at risk of not undergoing breast cancer screening in Spain. In our analyses, decision trees combined with an ensemble algorithm show that the importance of individual characteristics, such as education, marital status, or age, diverges based on the place of residence and their interaction. Particular attention ought to be placed on women living in specific regions: Ceuta and Melilla, single or legally separated women living in the rest of Spain, women not born in Spain, not residing in Ceuta or Melilla who are married, divorced, or widowed, and finally, illiterate women in lower social classes who are living in Asturias, Cantabria, Basque Country, Castile and León, Extremadura, Galicia, Madrid, Murcia, La Rioja, or Valencian Community at the protective intersection with marital status, age, and origin.

Beyond the Spanish context, applying a quantitative intersectionality framework that applies a comprehensive set of individual, system, and environmental indicators of inequality can greatly assist in the creation of health prevention programs that strive for equitable access to healthcare services.

## Data availability statement

The original contributions presented in the study are included in the article/supplementary material, further inquiries can be directed to the corresponding author.

## Ethics statement

Ethical approval was not required for the study involving humans in accordance with the local legislation and institutional requirements. Written informed consent to participate in this study was not required from the participants or the participants’ legal guardians/next of kin in accordance with the national legislation and the institutional requirements.

## Author contributions

NP: Conceptualization, Data curation, Formal analysis, Investigation, Methodology, Visualization, Writing – original draft, Writing – review & editing. BS: Conceptualization, Supervision, Validation, Writing – review & editing.
